# Improving HCV Screening in Addiction Care Centers with Plasma Separation Cards

**DOI:** 10.3390/pathogens14030239

**Published:** 2025-03-01

**Authors:** Fernando Velásquez Orozco, David Tabernero, María Gabriela Barbaglia, Lara Treviño, Begoña Trujillo, Andrés Marco, Miguel Ángel Carrillo, Gerard Ruiz Salinas, Francesc Xavier Majo Roca, Joan Colom Farran, María Buti, Tomas Pumarola-Sunyer, Francisco Rodriguez-Frias, Ariadna Rando-Segura

**Affiliations:** 1 Microbiology Department, Vall d’Hebron Hospital Universitari, Vall d’Hebron Barcelona Hospital Campus, Passeig Vall d’Hebron 119-129, 08035 Barcelona, Spain; tomas.pumarola@vallhebron.cat; 2 Departments of Genetics and Microbiology, Universitat Autònoma de Barcelona, Bellaterra, 08193 Barcelona, Spain; 3 Surgery and Surgical Department, Hospital Universistari del Mar, 08003 Barcelona, Spain; 4 Liver Unit, Vall d’Hebron Institut de Recerca (VHIR), Vall d’Hebron Hospital Universitari, Vall d’Hebron Barcelona Hospital Campus, Passeig Vall d’Hebron 119-129, 08035 Barcelona, Spain; david.tabernero@ciberehd.org (D.T.); mariaasuncion.buti@vallhebron.cat (M.B.); 5 Centro de Investigación Biomédica en Red de Enfermedades Hepáticas y Digestivas (CIBERehd), Instituto Carlos III, 28029 Madrid, Spain; frarodri@gmail.com; 6 Agència de Salut Pública de Barcelona, Pl Lesseps 1, 08023 Barcelona, Spain; mgbarbag@aspb.cat; 7 CAS Garbivent, 08027 Barcelona, Spain; lmtrevino@fvb.cat (L.T.); ext_btrujillo@aspb.cat (B.T.); 8 CAS Nou Barris, 08033 Barcelona, Spain; andres.marco.m@gmail.com (A.M.); mcarrillo@salut.abd.ong (M.Á.C.); 9 Centro de Investigación Biomédica en Red de Epidemiología y Salud Pública (CIBERESP), Instituto Carlos III, 28029 Madrid, Spain; 10 Biochemistry Department, Vall d’Hebron Hospital Universitari, Vall d’Hebron Barcelona Hospital Campus, Passeig Vall d’Hebron 119-129, 08035 Barcelona, Spain; gerardmanel.ruiz@vallhebron.cat; 11 Programme on Addictions, HIV, STI and Viral Hepatitis, Public Health Agency of Catalonia, Department of Health, Government of Catalonia, 08005 Barcelona, Spain; xavier.major@gencat.cat (F.X.M.R.); joan.colom@gencat.cat (J.C.F.); 12 Internal Medicine Department, Vall d’Hebron Hospital Universitari, Vall d’Hebron Barcelona Hospital Campus, Passeig Vall d’Hebron 119-129, 08035 Barcelona, Spain; 13 Department of Basic Sciences, Universitat Internacional de Catalunya (UIC), 08017 Barcelona, Spain

**Keywords:** hepatitis c infection, intravenous drug users, finger prick

## Abstract

Globally, 50 million people are infected with hepatitis C virus (HCV), many of whom are people who inject drugs. These individuals face healthcare barriers, necessitating innovative diagnostic tools. This study evaluated the impact of cobas plasma separation cards (PSCs) for dry plasma collection in Barcelona’s outpatient drug addiction centers (CAS). From February to December 2021, nine CASs were invited to implement PSC for HCV screening; three centers participated, allowing for the assessment of its impact on HCV detection. Of the 679 clients screened, 54 (8%) provided finger-prick blood samples via PSC due to their refusal or inability to undergo venipuncture. Overall, 100 (14.7%) clients tested positive for HCV antibodies, with 24 (24%) confirmed as HCV-RNA positive. Among venipuncture clients, 9.1% had positive antibodies, with 15.8% showing active infection. In contrast, 79.6% of PSC clients had positive antibodies and 34.9% had detectable HCV RNA, contributing to 62.5% of the active infections detected. The odds ratio was 26.3, indicating that refusal or inability to undergo venipuncture correlated with a significantly higher burden of active HCV infection. The findings highlight PSC as a valuable alternative for diagnosing HCV in people with substance use disorders, addressing accessibility barriers and improving linkage to care in high-risk populations.

## 1. Introduction

According to recent estimates, 50 million people were living with hepatitis C (HCV) by 2022 [1]. In 2016, the World Health Organization (WHO) established the goal of eliminating HCV by 2030. This ambitious target requires a significant reduction in the new cases of HCV infection and HCV-related deaths by 80% and 60%, respectively. Increasing diagnosis rates is one of the WHO’s objectives to achieve viral hepatitis elimination [2,3]. However, the number of newly diagnosed hepatitis C cases reported from countries across Europe remains at a high level. A recent systematic review estimates that 3.9 million individuals are chronically infected with HCV in EU/EEA countries [4]. The annual average incidence rate is estimated at 6.19 per 100,000 inhabitants (95% CI 4.90–7.48), with national estimates of anti-HCV prevalence in the general population ranging from 0.003 to 4.5% as reported by the WHO for the wider European region [5].

Despite efforts in screening programs, circuit improvements, harm reduction programs, and treatment with new direct-acting antiviral drugs, it is estimated that at least 40% of the infections are still undetected, and modeling suggests that morbidity and mortality will continue to increase [5]. The current profile of the undiagnosed patient is men aged 25–44 years, consistent with the demographic profile of individuals who inject drugs (PWIDs). The burden of infection remains high among PWIDs, with prevalence rates in some European series ranging from 50% to 83.2%. This alarming prevalence, coupled with evidence of ongoing transmission, emphasizes the need for targeted strategies for this at-risk population [6]. Identifying and overcoming barriers to screening this group is essential to fulfill the HCV elimination objective.

Data from the Centre for Epidemiological Studies on HIV/AIDS and STIs of Catalonia (CEEISCAT) and the HepCdetect II project indicate a prevalence of HCV among PWIDs of 65.8% and 58.9%, respectively [7]. In a prior study conducted by our team at the Vall d’Hebron outpatient drug addiction care and follow-up center (CAS), we observed that 19.2% of clients declined HCV testing, with 9.5% specifically refusing venipuncture [8]. Barriers such as the stigma associated with HCV testing, previous unsuccessful HCV treatments with interferon-based therapies [9], or resistance to venipuncture may significantly influence individuals’ willingness to undergo conventional screening. Overcoming these barriers requires, among other measures, the implementation of new diagnostic tools to improve access to screening.

Dried blood samples (DBSs) are an alternative method of sample collection that does not require venipuncture and is increasingly used to facilitate access to nucleic acid amplification tests (NAATs) and serological testing for HIV, hepatitis B and C, and other infectious diseases [10]. A device for collecting and stabilizing dried plasma from whole blood, the cobas^®^ Plasma Separation Card, has been commercialized. It shares many features with DBSs but offers advantages such as higher volume capacity, ease of transportation and storage, and an advanced stabilization membrane for storing and transporting samples under extreme heat and humidity.

The aim of this study was to evaluate the impact of introducing a new sample collection system, based on cards for the separation and stabilization of dry plasma (DPS), in the screening tools of the CAS attended by the clinical laboratories of the Vall d’Hebron Barcelona Hospital Campus.

## 2. Material and Methods

### 2.1. Clinical Setting

The clinical laboratories of the Vall d’Hebron Barcelona Hospital Campus serve nine CASs in the city of Barcelona, representing a significant coverage of the city, as they account for nine out of fifteen CASs. These centers offer comprehensive harm reduction programs, including consumption rooms. To increase access to HCV testing in CASs, we incorporated dried plasma spot (DPS) samples as an alternative to serum/plasma in the diagnosis of HCV. The performance of virological and serological HCV assays using DPS specimens was previously evaluated by our group [11]. DPS showed 92.8% sensitivity and 100% specificity for detecting anti-HCV antibodies and 94.9% sensitivity and 100% specificity for detecting HCV RNA [11].

### 2.2. Intervention

After a short nursing training course focused on the collection, preparation, and storage of DPS samples, we incorporated the HCV antibody and the HCV RNA in DPS in the laboratory test catalog of the nine CASs attended. A DPS card procurement flowchart template was designed (clinical laboratories centralized them and supplied them on demand). Participation in this intervention was voluntary for the CAS. Although all centers were invited to participate and attended the training, only three centers implemented the use of DPS cards. The specific reasons why the remaining six centers did not adopt the DPS methodology are unknown, although it is possible they did not encounter patients refusing venipuncture or faced implementation difficulties. All clients accessing CASs are routinely screened for HCV infection by venipuncture. Clients attending the participation centers during the period of the study who refused venipuncture and/or did not attend a previous appointment with the blood collection service were eligible for DPS sampling. All RNA-positive patients were referred to specialist care.

### 2.3. DPS Samples

DPS samples were collected by finger prick using Plasma Separation Card (Roche Diagnostics, Basel, Switzerland). Spots from DPS cards were dried at room temperature and kept in individual zipped plastic bags with a desiccant. DPS samples were stored at room temperature before being sent to the clinical laboratories of the Vall d’Hebron Barcelona Hospital Campus weekly (delivery to the laboratory using pre-existing specimen transport system). The response time of these samples was 24–48 h, and the processing of DPS was as previously described [11].

### 2.4. Data Inclusion Criteria

Only data from the CASs that voluntarily incorporated the DPS methodology were included in the analysis. CASs that did not adopt the DPS procedure were excluded from the analysis. It is important to acknowledge that differences in the populations served by the various CASs might introduce potential confounders.

### 2.5. Statistical Analysis

Descriptive statistics (percentage, median, interquartile range) were calculated for the main variables. Missing data were excluded from the analysis. The data were then analyzed at the bivariate level. A two-sample proportion test was used to compare categorical variables, and Student’s t-test was applied for continuous variables between clinical groups. Additionally, the odds ratio (OR) was directly calculated from a 2 × 2 contingency table, based on the observed data of exposure (screening subgroup) and outcome (HCV RNA-positive). The standard formula OR=ad/bc was used, where *a*, *b*, *c*, and *d* represent the frequencies of the data in the 2 × 2 table. No additional regression models were applied. All analyses were performed using Stata 12 (StataCorp LLC, College Station, TX, USA).

### 2.6. AI-Assisted Writing

During the preparation of this work, the author(s) used LiverAI 3.0 in order to improve the clarity and readability of the text. After using this tool/service, the author(s) reviewed and edited the content as needed and take(s) full responsibility for the content of the publication.

### 2.7. Ethical Considerations

This study was approved by the Research Ethics Committee of Vall d’Hebron Barcelona Hospital Campus. The approval code is PR(AG)528/2019, and the approval date was 7 February 2020. The study followed Good Clinical Practice guidelines, and all data were processed confidentially in an anonymous database in accordance with Spanish legislation.

## 3. Results

From February 2021 to December 2021, three of the nine CASs voluntarily adopted the DPS procedure for HCV screening. A total of 679 clients were screened for HCV in these three centers. Most were male (n = 523, 77.4%), and the mean age was 43.5 years (SD = 11.3). In 54 (54/679; 8.0%) cases, the blood sample was obtained by finger prick, as shown in Table 1. No statistically significant differences were observed in terms of sex (male gender, 76.9% screening by finger prick vs. 83.0% screening by venipuncture), *p* = 0.2249. But patients screened by venipuncture were younger than patients screened by finger prick (42.7y, SD = 11.3; vs. 52.6y, SD = 7.4), *p* < 0.05. Among the three centers using DPS cards, the first accounted for 79.6% of the 43 screenings, while the third used DPS cards for only one patient (1.9%). This highlights the differing adoption rates of DPS cards among the centers, suggesting differences in center practices or patient populations.

### 3.1. Hepatitis C Infection

Overall, 100 (100/679; 14.7%) HCV antibody-positive individuals were identified, as shown in Table 2, of whom 24 (24/100; 24%) were HCV RNA-positive, as shown in Table 3. Among the clients who accepted the venipuncture screening, 57 (57/625; 9.1%) people with positive antibodies were identified, of which nine (9/57; 15.8%) were positive for HCV-RNA. Among clients who accepted finger prick screening, 43 (43/54; 79.6%) people with positive antibodies were identified, of which 15 (15/43; 34.9%) were HCV RNA-positive.

Of the HCV antibody-positive individuals (Table 2), most were male (n = 80, 80%), and the mean age was 50.1 years (SD = 7.9). In 43 (43/100, 43%) cases, the blood sample was obtained by finger prick. No statically significant differences were observed in terms of sex (*p* = 0.8430) and age (*p* = 0.1179).

Of the HCV RNA-positive individuals (Table 3), most were male (n = 19, 79.2%), and the mean age was 47.8 years (SD = 8.3). In 15 (15/24, 62.5%) cases, the blood sample was obtained by finger prick. No statistically significant differences were observed in terms of sex (*p* = 0.3624). However, patients screened by venipuncture were younger than those screened by finger prick (42.8 years, SD = 7.2; vs. 50.5 years, SD = 7.7), *p* = 0.0297. No statistically significant differences were observed in terms of liver fibrosis, although among clients who accepted finger prick screening, we found a higher percentage of patients with a FIB-4 > 3.25, corresponding to severe fibrosis (F3 to F4), as shown in Table 3.

Additionally, the odds ratio (OR) was directly calculated from a 2 × 2 contingency table based on the observed data of exposure (screening subgroup) and outcome (HCV RNA-positive). Patients screened using dried blood samples (finger prick) are 26.3 times more likely to test positive for HCV RNA compared to those screened via venipuncture (OR = 26.3).

### 3.2. Hepatitis C Cascade of Care

**Figure 1:**
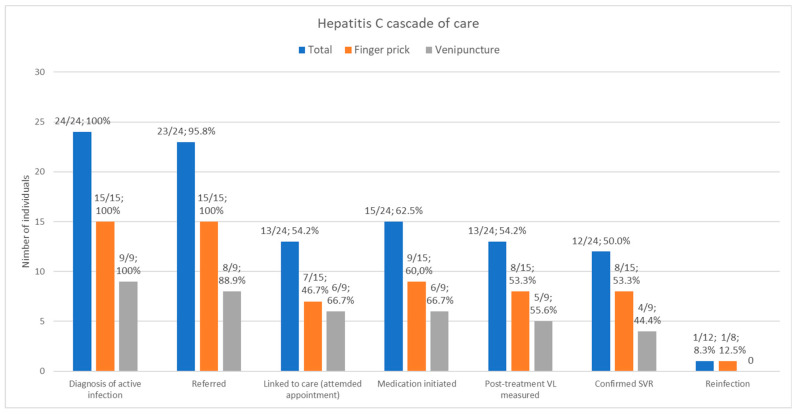
Hepatitis C cascade of care. VL, viral load; SVR, sustained virologic response.

Twenty-four patients were diagnosed with active HCV infection, twenty-three were referred to a specialist (23/24; 95.8%), but only thirteen attended the appointment (13/24; 54.2%). Out of the ten patients who were not initially linked to treatment, two were subsequently initiated on therapy. One patient started treatment 1.5 years later following an emergency room admission due to spontaneous bacterial peritonitis (SBP) as a complication of liver cirrhosis by HCV that required a liver transplant. The other one was linked to treatment 2.6 years after at an HIV care clinic. All patients linked to care were treated (15/24; 62.5%). The end treatment response was 100% (two patients were not tested because they did not attend the visit), and sustained virological response (SVR) was also 100% (three patients were not tested because they had not attended the visit), as shown in Figure 1.

One case of HCV reinfection after successful treatment was detected (DPS subgroup).

## 4. Discussion

The study encompassed 679 participants, predominantly male (77.4%), with an average age of 43.5 years (SD = 11.3 years). Of this cohort, 14.7% were found to be HCV antibody-positive, and within this seropositive group, 24% (24 individuals) were also HCV RNA-positive, indicating active infection. The venipuncture screening subgroup had a lower prevalence of antibody positivity (9.1%) compared to the finger prick subgroup (79.6%), with 15.8% and 34.9%, respectively, being HCV RNA-positive. The HCV RNA-positive cohort maintained similar demographic characteristics to the overall study population, with a male majority and a mean age of 47.8 years (SD = 8.3). While no significant sex distribution difference was observed between the two sampling methods (*p* = 0.3624), a significant age difference was noted, with venipuncture-screened patients being younger than finger prick-screened individuals (42.8 years vs. 50.5 years, *p* = 0.0297). Additionally, 25% of the patients diagnosed through finger prick had a FIB-4 score greater than 3.25, indicative of advanced liver fibrosis, while no patients screened by venipuncture had FIB-4 scores above this threshold. Despite the limited sample size and incomplete FIB-4 data, these findings suggest a higher risk of advanced liver disease in the finger prick subgroup.

The Spanish seroprevalence study shows the highest prevalence of HCV in the 50-to-59-year age group [12]. In contrast, the European Centre for Disease Prevention and Control (ECDC) reports identify the 35-to-44-year age group as the highest-risk group [13]. This discrepancy is due to demographic differences and risk factors between the populations studied. Spanish data were obtained from a population-based study with a low representation of vulnerable groups [12]. while the ECDC data focus on high-risk groups, especially PWIDs [13].

In our study, venipuncture screening identified a younger cohort of viremic patients, aligning more with ECDC data. Conversely, DPS screening detected older patients, corresponding to the highest seroprevalence age group in Spain. A critical limitation of our study is the lack of data on the socioeconomic and psychosocial factors of the screened patients. This prevents us from understanding the differential characteristics of these two groups. Barriers such as the stigma associated with HCV testing or undertaken HCV treatment unsuccessfully with previous interferon-based therapies may significantly influence individuals’ willingness to undergo conventional screening [9].

CASs provide integral care for individuals who consume legal and illegal drugs [14]. The overall prevalence of HCV antibodies and active infection was observed to be 14.7% and 3.5%, respectively. These rates are significantly lower compared to the prevalence reported by the CEEISCAT and the HepCdetect II project, which specifically focus on PWIDs, where the prevalence is 65.8% and 58.9%, respectively [7]. However, when focusing on the subgroup of individuals who either refused venipuncture or did not attend scheduled blood collection, the detected prevalence of HCV antibodies and active infection increased to 79.6% and 27.8%, respectively. Moreover, although only a small fraction of the total screened patients (54 out of 679, or 8.0%) were tested using DPS samples, 62.5% (15 out of 24) of all active HCV infection diagnoses were made in the DPS subgroup. These findings indicate that patients who opted for finger prick screening in our study demonstrate a prevalence of HCV antibodies similar to that observed in PWIDs [15]. This suggests DPS is particularly effective in capturing self-selected high-risk populations, potentially comparable to PWIDs in terms of HCV exposure. However, the lack of data on the socioeconomic and psychosocial factors of the screened patients may limit the generalizability of the results, as the identified individuals are not necessarily typical of all PWIDs, although they do represent a large fraction of active infection diagnoses.

One of the most alarming findings of this study is that only 54.2% (13 out of 24) of the diagnosed patients initiate treatment, highlighting a significant gap in the care continuum. Without ensuring this crucial connection, the impact of diagnostics efforts is greatly diminished. Current strategies appear insufficient in linking this patient subset to the strategies that facilitate access to treatment at the point of care, which may help bridge the gap in necessary care. Therefore, implementing this bridge is necessary [16,17].

This study has several limitations. The small sample size of RNA-positive cases and the lack of data on the socioeconomic and psychosocial factors of the screened patients may limit the generalizability of the results. Additionally, the absence of complete FIB-4 data and other liver health measures may affect the interpretation of findings related to hepatic fibrosis.

Furthermore, participation in the DPS procedure was voluntary, and only three out of the nine centers implemented it despite receiving invitations and training. The reasons for non-adoption by the other six centers are unclear and may include a lack of eligible patients or implementation challenges. This selective adoption could introduce bias, as centers that participated might differ from those that did not, affecting the representativeness of our findings.

However, we believe that the DPS should be available in all outpatient drug addiction treatment centers. Despite the workload involved in processing this type of sample and the fact that the preferred sample for HCV screening is plasma obtained by venipuncture, DPS can serve as a primary diagnostic tool in centers that do not have blood draw services or as an effective alternative for those who refuse venipuncture or miss appointments.

## 5. Conclusions

Individuals who refuse HCV infection screening via venipuncture are at a significantly increased risk of having an active HCV infection (OR = 26.3) (27.8% [15/54] versus 1.4% [9/625]). Notably, 62.5% of active infection diagnoses were made in people who were offered finger prick screening.

Implementing more accessible screening strategies, such as the use of DPS samples, could significantly enhance the detection and treatment of HCV in high-risk populations. We recommend that public health policies consider integrating these strategies into their HCV control programs.

Achieving the goal of eradicating HCV in high-risk populations requires not only accurate diagnosis but also effective linkage to treatment. It is imperative to explore methods to enhance linkage to care, ensuring that individuals diagnosed with HCV receive the necessary treatment.

Finally, collecting detailed information on socioeconomic and psychosocial factors is crucial for understanding the underlying dynamics and developing more targeted and effective screening and treatment strategies, improving uptake across all at-risk groups.

## Figures and Tables

**Table 1:** Baseline characteristics of clients screened for HCV using different blood sampling methods.

	Overall (n = 679)	Finger Prick (n = 54)	Venipuncture (n = 625)
Age (years), mean (95%CI)	43.5 (42.6–44.3) (47 missing data)	52.6 (50.5–54.7) (4 missing data)	42.7 (41.8–43.6) (43 missing data)
Stratified by *sex* *			
Female	22.6% (19.6–26.0)	17.0% (9.0–29.7)	23.1% (20.0–26.6)
Male	77.4% (74.5–80.4)	83.0% (70.3–91.0)	76.9% (73.4–80.0)
Stratified by *center*			
Center 1	43.4% (39.8–47.2)	79.6% (66.7–88.4)	40.3 (36.5–44.2)
Center 2	31.8% (28.4–35.4)	18.5% (10.2–31.3)	33.0 (29.4–36.8)
Center 3	24.7% (21.6–28.1)	1.9% (0.3–12.2)	26.7 (23.4–30.3)

* Two missing data (one screening venipuncture and one finger prick).

**Table 2:** Baseline characteristics of individuals with anti-HCV antibodies.

	Overall (n = 100)	Finger Prick (n = 43)	Venipuncture (n = 57)
Age (years), mean (95%CI)	50.1 (48.5–51.7) (10 missing data)	51.6 (49.1–53.8) (3 missing data)	48.9 (46.7–51.2) (7 missing data)
Stratified by *sex* *			
Female	19.0% (11.8–28.1)	20.9% (10.0–36.0)	17.5% (8.7–29.9)
Male	80.0% (70.8–87.3)	79.1% (64.0–90.0)	80.7% (68.1–90.0)
Stratified by *center*			
Center 1	65.0% (55.1–73.7)	84.1% (70.0–92.3)	50.0% (37.1–62.9)
Center 2	21.0% (14.1–30.1)	15.9% (7.7–300)	25.0% (15.3–38.1)
Center 3	14.0% (8.4–22.3)	0.0%	25.0% (15.13–37.8.1)

* One missing data (screening venipuncture).

**Table 3:** Baseline characteristics of HCV RNA-positive individuals.

	Overall (n = 24)	Finger prick (n = 15)	Venipuncture (n = 9)
Age (years), mean (95%CI)	47.8 (44.2–51.4) (1 missing data)	50.5 (46.2–54.8) (No missing data)	42.8 (36.8–48.7) (1 missing data)
Male gender, n (%)	19 (79.2%) (No missing data)	11 (73.3%) (No missing data)	8 (88.9%) (No missing data)
Platelets (×10^9^/L), median (range)	186 (79–385) (3 missing data)	138.5 (79–355) * (3 missing data)	249 (142–385) (No missing data)
AST (IU/L), median (range)	46 (23–306) (3 missing data)	44 (23–112) * (3 missing data)	67 (25–306) (No missing data)
ALT (IU/L), median (range)	47 (11–594) (3 missing data)	40.5 (11–65) * (3 missing data)	66 (27–594) (No missing data)
FIB-4 > 3.25, n (%)	3 (14.3%) (4 missing data)	3 (25.0%) (4 missing data)	0 (0.0%) (1 missing data)
APRI > 1.5, n (%)	5 (25%) (3 missing data)	2 (18.2%) (3 missing data)	3 (33.3%) (No missing data)

* Data not available (these markers cannot be performed in dried blood samples). The data correspond with baseline screening previously to treatment.

## Data Availability

The original contributions presented in this study are included in the article. Further inquiries can be directed to the corresponding authors.
